# The costs of integrated community case management (iCCM) programs: A multi–country analysis

**DOI:** 10.7189/jogh.04.020407

**Published:** 2014-12

**Authors:** David Collins, Zina Jarrah, Colin Gilmartin, Uzaib Saya

**Affiliations:** Management Sciences for Health, Medford, MA, USA

## Abstract

**Background:**

Integrated community case management (iCCM) can be an effective strategy for expanding the provision of diarrhea, pneumonia, and malaria services to children under 5 years old but there are concerns in some countries about the corresponding cost and impact. This paper presents and compares findings from a multi–country analysis of iCCM program costs.

**Methods:**

Data on coverage, utilization, and costs were collected as part of two sets of studies conducted between 2011 and 2013 for iCCM programs in seven sub–Saharan African countries: Cameroon, the Democratic Republic of the Congo, Malawi, Senegal, Sierra Leone, South Sudan and Zambia. The data were used to compare some elements of program performance as well as costs per capita and costs per service (which are key indicators of resource allocation and efficiency).

**Results:**

Among the seven countries, iCCM utilization ranged from a total of 0.26 to 3.05 contacts per capita (children 2–59 months) per year for the diseases treated, representing a range of 2.7% to 36.7% of the expected numbers of cases. The total recurrent cost per treatment ranged from US$ 2.44 to US$ 13.71 for diarrhea; from US$ 2.17 to US$ 17.54 for malaria (excluding rapid diagnostic testing); and from US$ 1.70 to US$ 12.94 for pneumonia. In some of the country programs, the utilization of iCCM services was quite low and this, together with significant fixed costs, particularly for management and supervision, resulted in services being quite costly. Given the differences across the countries and programs, however, these results should be treated as indicative and not definitive.

**Conclusion:**

A comprehensive understanding of iCCM program costs and results can help countries obtain resources and use them efficiently. To be cost–effective and affordable, iCCM programs must be well–utilized while program management and supervision should be organized to minimize costs and ensure quality of care. iCCM programs will not always be low–cost, however, particularly in small, remote villages where supervision and supply challenges are greater. Further research is needed to determine the cost–effectiveness of iCCM programs and corresponding patient and service delivery costs.

Due to limited access to effective treatment, diarrhea, malaria and pneumonia remain the leading causes of child mortality in sub–Saharan Africa and result in nearly 41% of global deaths in children under five years old [1]. To improve access to treatment of these illnesses, several developing countries have adopted integrated community case management (iCCM) – the delivery of timely interventions at the community level by community health workers (CHWs). This is seen as a key strategy in meeting Millennium Development Goal 4 on reducing child mortality by 2015.

To be effective, iCCM services must be available from a single provider (“one–stop shopping”) within 24 hours of the onset of symptoms. For example, if a child has a fever, the parent should be able to see a CHW in his or her community within 24 hours and the CHW should be able to provide diagnosis and treatment if the case is simple and refer the case if it is not. The integration of these services is important – there is growing evidence that this increases the utilization of malaria and pneumonia treatment [2–4] compared with separate community–based interventions, and also delivers more timely and appropriate treatment for fever, including malaria. Easy access is also crucial and the availability of iCCM services is especially important in hard–to–reach areas where people live far from health facilities.

Despite the reported success of iCCM in several low– and middle–income countries, it has yet to be implemented as a national strategy in some other countries. This is partly due to concerns about the costs and financing of iCCM programs and the justification of the extra investment in terms of the related health outcomes. A comprehensive understanding of the costs and results will help countries who are considering implementing or expanding iCCM programs to advocate for funding and to plan and budget appropriately. It will also allow for costs to be better monitored and controlled, thus contributing to the efficient use of scarce resources.

This paper describes and compares the results of iCCM cost analyses conducted under two separate sets of studies in seven sub–Saharan African countries.

## METHODS

The cost analyses were conducted between 2011 and 2013. The first two studies were conducted of national programs in Malawi and Senegal in 2011 and 2012 as part of the testing of an iCCM costing and financing tool under the United States Agency for International Development (USAID) Translating Research into Action Project, and these countries were selected because they have mature iCCM programs and sufficient data. A third study conducted in Rwanda was excluded because data were not comparable. The second set of five studies was conducted of sub–national programs in Cameroon, Democratic Republic of Congo (DRC), Sierra Leone, South Sudan, and Zambia in 2013 with funding from the Bill and Melinda Gates Foundation (BMGF). These five countries were selected by BMGF to estimate the costs of five iCCM projects funded by another international donor. The areas where these five studies were conducted were, reportedly, based on need and feasibility, and all included areas with hard–to–reach populations.

Data were obtained from records and through interviews with the CHWs who provided iCCM services, their supervisors, and program managers. A standard questionnaire was used for the interviews. The samples of districts, health centers, and communities were selected purposefully in terms of access and availability of health facility staff and CHWs. Time limitations and access constraints, such as poor road conditions, meant that some samples may not have included health facilities and CHWs from very remote areas. The samples were relatively small but were sufficient to validate service delivery protocols and to collect data on the work and supervision of the CHWs. An average of 12 health centers were visited in each country, and interviews were conducted with an average of three CHWs per health center, totaling approximately 36 CHWs in each country.

The costs were analyzed using the USAID iCCM Costing and Financing Tool (the tool is available at www.msh.org/iccm and is described in detail in the individual country studies). At the service delivery level, this is a bottom–up, activity–based costing tool, in which standard costs are used to estimate total direct costs per service. Indirect costs, such as supervision and training, are then allocated based on CHW time estimates using a top–down methodology. The resulting figures are a mixture of standard and actual costs, obtained from accounting and budget records and through interviews, in what is sometimes known as an “ingredients” approach [5]. The costs shown were generally total costs incurred by both governments and NGOs and financed from government and donor sources.

The data collection and initial analysis took an average of three weeks in–country involving a small team of experienced data collectors and one experienced health economist. The final analysis, report–writing and validation for each study took an additional two weeks.

### Country studies

All seven iCCM programs varied in terms of study period, population density and coverage, incidence rates, hard–to–reach populations, the nature of the implementing organization (government or NGO), supervision, supply chain, CHW remuneration, user fees, and other aspects. These many differences limit the usefulness of direct comparisons of the findings, but add richness to the analysis. More information on the country studies can be found in the individual country reports [6–13] – brief descriptions are below:

**Malawi**’s national iCCM activities began in 10 districts in 2008 and, with support from donors, were scaled–up throughout the country by 2010. CHWs were remunerated by the government and were expected to spend two days per week on iCCM activities at village clinics and to participate in active case finding through household visits. iCCM services comprised the treatment of malaria, diarrhea, pneumonia, and red eye as well as the identification and referral of anemia and malnutrition. The costing study was based on a sample of iCCM services in the 2328 hard–to–reach communities covered by iCCM.

**Senegal**’s iCCM program started in 2003. Services were provided through USAID’s Community Health Program which covered the whole country in collaboration with the Ministry of Public Health (MoPH). Services were provided at health huts in the communities and were meant to cover rural, remote areas that did not have health posts (the lowest level of facility). The iCCM service package included rapid diagnostic tests (RDTs) and malaria treatment, and diarrhea and pneumonia treatment. User fees were charged and patients were supposed to purchase the medicines; the prices included a mark–up of 5% to 25%. The funds were intended to be used to replenish stocks and to cover other costs.

In **Cameroon**, a local NGO, in collaboration with the MoPH, began implementing an iCCM project in 2009 in two remote districts – Nguelemendouka and Doumé. Through this project, volunteer CHWs provided free treatment to children between the ages of 2 to 59 months for cases of malaria and diarrhea. Treatment for pneumonia was added in Nguelemendouka District in 2013.

In the **Democratic Republic of the Congo** (DRC), implementation of a national iCCM program began in 2005 under the leadership of the MoPH. In 2010, iCCM services were expanded to include family planning services, and the MoPH mandated that malaria treatment be integrated at community sites, along with pneumonia and diarrhea treatment services. The focus of the costing study was the iCCM component of a project in 9 of the 16 health zones of the Sud–Ubangi District in Equateur Province. The project started in 2010 and was implemented by a local NGO in coordination with the MoPH.

In **Sierra Leone**, an international NGO led an iCCM project which began in Kono district in May 2006. Unpaid Community Health Volunteers provided free treatment to children ages 2 to 59 months for malaria, diarrhea, and pneumonia. Starting in September 2013, the plan was to expand the role of the CHW to include the delivery of community–based maternal and newborn health care interventions.

In **South Sudan**, an international NGO began implementation of an iCCM program in 2009 in hard–to–reach areas in five states and ten counties. These include Kapoeta North County which was selected for the costing study. Unpaid Community–Based Distributors provided free treatment to children ages 2 to 59 months for cases of malaria, diarrhea, and pneumonia.

In **Zambia,** volunteer CHWs began conducting iCCM activities in four districts of Luapula Province in late 2010, then scaled up to all seven districts in 2012, serving a population of 741 373 in remote communities. The iCCM package included RDTs and treatment of pneumonia, diarrhea and malaria, and was implemented in areas where access to health facilities and services was limited. The program had a demand generation element, including having CHWs conduct behavior change communication activities. The project was managed by an international NGO working closely with the Ministry of Health (MoH) as part of a national iCCM program being implemented by the MoH across the country, although this project may not have been completely representative of the overall MoH program.

A summary of key elements of the iCCM programs studied are shown in **Table 1**.

**Table 1:** Geographical and population coverage of iCCM programs covered by the costing studies

	National programs		Sub–national programs				
	**Malawi**	**Senegal**	**Cameroon**	**DRC**	**Sierra Leone**	**South Sudan**	**Zambia**
Year when iCCM implementation started	2008	2003	2009	2010	2006	2009	2010
Year of utilization and cost data	2010	2011	2012	2012	2012	2012	2011
Number of districts (or equivalent) covered	28	65	2	9	14	10	4
Number of health centers supervising iCCM	400	1061	20	126	68	48	77
Total number of communities covered by iCCM	10 451	1620	213	396	900	313	684
Number of hard–to–reach communities covered by iCCM	2328	1620	213	396	900	313	684
Number of CHWs providing iCCM	2328	3240	419	805	840	891	720
iCCM catchment population 2–59 months	615 149	239 860	25 114	138 100	31 584	160 004	78 797
Average number of children 2–59 months per CHW	454	74	60	171	38	180	109

iCCM – integrated community case management, CHW – community health worker, DRC – Democratic Republic of the Congo

## RESULTS

### Coverage and utilization

The package of iCCM services varied across the programs, with only six of the seven covering the three illnesses in an integrated way (**Table 2**). In Cameroon pneumonia treatment was not part of the package at the time of the study. In some cases, more services were included; for example, treatment of red eye and anemia in Malawi. Malaria treatment was provided symptomatically for fever in Malawi, Cameroon, DRC, Sierra Leone and South Sudan, whereas RDTs were used to detect malaria in Senegal and Zambia. Based on estimates of incidence, the expected number of total annual episodes of illness per child (2–59 months) in the programs where the three main diseases were covered ranged from 5.3 in Malawi to 9.6 in Senegal (where fever was included in the number of episodes, we excluded the numbers of malaria episodes to avoid double–counting). The catchment areas comprised hard–to–reach communities, and it was assumed that there was no access to health facilities or other qualified service providers and all cases should, therefore, have been seen by CHWs. This may have resulted in an overestimate in terms of expected numbers of diarrhea cases to be treated by CHWs, since home treatment using oral rehydration therapy has been taught and promoted in some communities for many years.

**Table 2:** Number of cases per capita (children aged 2–59 months) in the study year*

	National programs		Sub–national programs				
	**Malawi (2010)**	**Senegal (2011)**	**Cameroon (2012)**	**DRC (2012)**	**Sierra Leone (2012)**	**South Sudan (2012)**	**Zambia (2011)**
Fever treated presumptively as malaria	0.52	0	0.40	0.90	1.59	0.41	0
Fever tested for malaria with RDT	0	0.10	0	0	0	0	1.62
Confirmed malaria cases treated	0	0.03	0	0	0	0	1.30
Pneumonia cases treated	0.33	0.10	0	0.53	0.55	0.29	0.40
Diarrhea cases treated	0.12	0.06	0.24	0.45	0.91	0.24	0.20
Other cases treated	0.04	0	0	0.14	0	0	0
Referrals made	NA	NA	NA	0.11	NA	0.01	0.38
Total services utilized per child (fever/malaria, pneumonia, diarrhea only)	0.97	0.26	0.64	1.88	3.05	0.94	2.22
Total expected cases per child based on estimated incidence (fever/malaria, pneumonia, diarrhea)†	5.3	9.6	6.1	8.9	8.3	6.5	6.7
Total cases treated as % of total expected cases	18.3%	2.7%	10.5%	21.1%	36.7%	14.5%	33.1%

DRC – Democratic Republic of the Congo, RDT – rapid diagnostic test, NA – not available

*We did not include the treatment of non–malaria fever as a separate service, although in some cases fever–reduction medication such as paracetamol is provided and there is, therefore, a cost.

†Sub–Saharan Africa incidence rates were used for all three services in Malawi [14–16] and for malaria and pneumonia in Rwanda [14,15].

The average total numbers of services provided per child per year ranged from 0.26 in Senegal to 3.05 in Sierra Leone and as percentages of the expected numbers of cases in the hard–to–reach areas ranged from 2.7% to 36.7% (also in Senegal and Sierra Leone). These comparisons should be treated as indicative, as estimating the catchment populations in the hard–to–reach areas was difficult. A major difference was the treatment of malaria (diagnosed or presumptive) which accounted for higher numbers of treatments in Zambia, Sierra Leone and, to some degree, in the DRC. Numbers of referrals were only available in DRC, South Sudan and Zambia and amounted to 0.11, 0.01, and 0.38 per child per year, respectively. In South Sudan and Zambia these figures translate to about 1% and 14% of total cases, respectively. A rule of thumb used by some providers is that around 10% of cases need to be referred – a referral rate that is too low may indicate that the provider is treating too many severe cases, whereas one that is too high may indicate a lack of medicines or supplies or a lack of confidence.

### Non–recurrent costs

The costs of starting an iCCM program can include the development of plans, policies, guidelines and training materials– most of which are generally financed by the national government and/or partners. For this study, we only took into account the training and equipping of CHWs (and in some cases, of their supervisors). All costs for these activities were included irrespective of who incurred or funded them. If the training included more health topics than iCCM, we only included the proportion related to iCCM. The start–up costs for training and equipment were mostly in the range from US$ 202 to US$ 352 per CHW, with Malawi and Zambia being outliers at US$ 1058 and US$ 897, respectively (these costs are in 2012 US$, representing the cost of training and equipment if it were provided in 2012). In Malawi, costs were higher because they included a portion of general CHW training and an incentive payment. In Zambia costs were higher because they included training–of–trainers and supervisors, the training was longer than in the other countries, and per diem rates were high relative to those in other countries.

The reported annual CHW attrition rates varied significantly across the study sites, ranging from 2% in Malawi (where they are remunerated) to an estimated 10% in South Sudan (the DRC figure of 40% was an unofficial estimate and may not be reliable). The costs of training and equipping replacement CHWs can be significant, as shown above, with most costs in the range of US$ 202 to US$ 352 per provider.

While it is possible to amortize non–recurrent costs over the expected period of use and include them with recurrent costs, this was not done here as it would have been difficult to estimate certain aspects, such as the length of the use of equipment or how long a CHW will work after the initial training.

### Recurrent costs

A comparison of recurrent costs can provide meaningful perspectives on the resourcing, equity, and efficiency of service provision and support, and can provide input into cost–effectiveness analyses, involving comparisons of costs per output or outcome. Recurrent costs are those repeated on an ongoing basis and, in this study, include medicines and supplies, management, supervision, and refresher training. These costs are expressed in two ways: per capita and per service. The costs of the training and equipping of replacement CHWs were not included in recurrent costs in these studies although it would be reasonable to do so.

Per capita recurrent costs are calculated here by dividing the total recurrent cost by the number of children in the catchment population. With the exception of the costs of medicines and supplies (which represent estimates of the quantities consumed), these figures represent the iCCM resources made available to the catchment populations. Per service recurrent costs, on the other hand, are calculated by dividing the total recurrent cost by the number of services provided. These figures represent the iCCM resources that should have been used in providing a single service. The ratio between per–capita and per–service costs is the same as the rate at which services are used per capita.

Medicines and supplies are variable costs which change based on the numbers of services provided. Provider remuneration, management, supervision, refresher trainings and other similar costs are generally fixed and do not vary with the number of services provided. It is important to note that the average total costs of medicines and supplies vary with the mix of services provided as well as with the unit costs of the various medicines. So if a greater proportion of services with higher–cost medicines is used, the average costs across all services will be higher.

The average total recurrent costs per capita (children aged 2–59 months) were much lower for the two national programs (Malawi and Senegal), which were US$ 2.16 and US$ 2.07, respectively, than for the four sub–national programs with the complete iCCM package (DRC, Sierra Leone, South Sudan and Zambia), which ranged from US$ 5.50 to US$ 10.20 (**Table 3**). This is largely due to economies of scale in the national programs where the fixed costs, especially of management and supervision, are spread across much higher catchment populations. As noted previously, however, caution should be used in comparing costs across the countries since there were many contextual differences.

**Table 3:** Recurrent costs by category (in US$)*

	National programs		Sub–national programs				
	**Malawi (2010)**	**Senegal (2011)**	**Cameroon (2012)**	**DRC (2012)**	**Sierra Leone (2012)**	**South Sudan (2012)**	**Zambia (2011)**
**Cost per capita (2–59 months)**							
Medicines and supplies	0.54	0.14	0.29	0.74	1.54	0.53	2.54
CHW remuneration	1.41	0	0	0	0	0	0
Management and supervision	0.07	1.62	8.40	4.39	8.65	6.90	6.90
Meetings	0.15	0.22	0.84	0.38	0	0.46	0.16
Refresher Training	0	0.09	0.75	0	0	0.68	0
**Total**	**2.16**	**2.07**	**10.26**	**5.50**	**10.20**	**8.58**	**9.60**
**Average cost per service**							
Medicines and supplies	0.53	0.47	0.46	0.34	0.50	0.56	0.65
CHW remuneration	1.40	0	0	0	0	0	0
Management and supervision	0.07	5.39	13.15	2.02	2.79	7.23	1.78
Meetings	0.15	0.74	1.32	0.17	0	0.48	0.04
Refresher Training	0	0.29	1.18	0	0	0.72	0
**Total**	**2.15**	**6.89**	**16.11**	**2.53**	**3.29**	**8.99**	**2.47**
**Cost breakdown %**							
Medicines and supplies	25%	7%	3%	13%	15%	6%	26%
CHW remuneration	65%	0%	0%	0%	0%	0%	0%
Management and supervision	3%	79%	82%	80%	85%	81%	72%
Meetings	7%	11%	8%	7%	0%	5%	2%
Refresher training	0%	4%	7%	0%	0%	8%	0%
	100%	100%	100%	100%	100%	100%	100%

DRC – Democratic Republic of the Congo, CHW – community health worker

*Addition errors in summary numbers are due to rounding.

The average total recurrent cost per service ranged from US$ 2.15 in Malawi to US$ 8.99 in South Sudan. Cameroon was an outlier at US$ 16.11 due to the high level of management and supervision costs combined with low utilization rates, taking into account that pneumonia treatment was not part of the iCCM package at the time of the study. There was no major difference between the national and sub–national costs per service. In general, lower costs per service were related to higher utilization levels combined with lower management and supervision costs. Differences in case mix did not seem to be major factors since the unit cost per disease followed a similar pattern.

The average cost per service for medicines and supplies ranged from US$ 0.34 in the DRC to US$ 0.65 in Zambia. Costs were higher in Zambia because RDTs were included. The mix of services and purchasing prices of medicines were different in each country so these figures are not directly comparable.

The iCCM portion of the salary payments to the CHWs in Malawi was significant at US$ 1.40 on average per service. We did not collect information on the user fees charged by the CHWs in Senegal, which is also a form of remuneration. CHWs were not formally remunerated in any of the 5 sub–national projects.

Management and supervision costs ranged from 72% to 85% of total recurrent costs among the sub–national programs compared with 3% for the national program in Malawi. The Malawi figure was proportionally lower because 65% of the total cost went on CHW remuneration. The Senegal figure of 79% was much higher than that of the Malawi programs because it was managed through a donor–funded project. A key factor in the high cost of supervision in South Sudan was that the implementing NGO had to supervise the CHWs and that was done from central levels as it could not be done from the health facilities. It is important to note that the variations in the way these management and supervision costs were captured, calculated, and reported mean that these comparisons across the programs are indicative and not definitive.

The costs of CHW meetings ranged from US$ 0.15 per capita in Malawi to US$ 0.84 in Cameroon (no separate cost was recorded for Sierra Leone). These costs depended mainly on the frequency of meetings, per diem rates, and amounts reimbursed to CHWs for transport. Refresher training was sometimes provided as part of the routine supervision system or through meetings – in others it was provided as a separate dedicated training activity. In the programs where it was a separate activity, the average cost per capita ranged from US$ 0.09 to US$ 0.75.

Recurrent costs can be more meaningfully compared by type of service (eg, malaria) since the average total costs across all services are affected significantly by variations in service mix. Diarrhea treatment was the only service provided in all the studies and the recurrent cost ranged from US$ 2.44 per service in Malawi to US$ 7.80 in South Sudan (**Table 4**). Pneumonia diagnosis and treatment costs ranged from US$ 1.70 in Malawi to US$ 12.94 in South Sudan. And the cost of presumptive malaria treatment ranged from US$ 2.17 in the DRC to US$ 7.10 in South Sudan. Cameroon was an outlier in these measurements because of the high support costs and low utilization level described earlier. It is important to note that the costs of treating presumptive malaria in some countries cannot be compared with the costs of testing and treating malaria in others because of the contextual differences among the countries.

**Table 4:** Total average recurrent cost for each type of service (in US$)

	National programs		Sub–national programs				
**Recurrent cost per service**	**Malawi (2010)**	**Senegal (2011)**	**Cameroon (2012)**	**DRC (2012)**	**Sierra Leone (2012)**	**South Sudan (2012)**	**Zambia (2011)**
Fever treated presumptively as malaria	2.38		17.54	2.17	3.27	7.10	
RDT testing for malaria		6.89					3.69
Malaria treatment		3.47					3.90
Pneumonia diagnosis and treatment	1.70	8.75		3.55	3.45	12.94	3.56
Diarrhea diagnosis and treatment	2.44	5.89	13.71	2.70	3.22	7.80	3.60
Referrals				1.10	1.08	3.87	1.50

DRC – Democratic Republic of the Congo, RDT – rapid diagnostic test

The cost per type of iCCM service depends on two important factors – the CHW’s time and the cost of medicines and supplies. The time that CHWs spend on providing each type of service was used to allocate their remuneration (if they were paid) and the indirect costs (eg, supervision) across the service types. The estimates of time used in the studies were obtained through CHW interviews and ranged widely, for example, from 26 minutes to 91 minutes for pneumonia diagnosis and treatment in Malawi and Sierra Leone, respectively. Such figures should be treated with caution as in some cases the CHWs had difficulty in understanding the concept of estimating the times spent in diagnosing and treating patients. They can also be affected by the way the time is estimated, for example, if a CHW includes the time taken to travel to the house of the patient. It is important to note that some CHWs reported making follow–up visits to patient’s homes and this was not generally taken into account.

The reported unit cost per episode for tests and medicines also varied across the study sites, for example ranging from US$ 0.04 to US$ 0.56 for pneumonia treatment in Senegal and Zambia, respectively (**Table 5**). These unit costs are affected by different treatment regimens and different procurement prices as well as the split in treatment doses among age groups. For example, the average cost per episode for medicines and tests for pneumonia was much lower in Senegal, Malawi and DRC, where Cotrimoxazole was used, compared to Zambia, where Amoxicillin was used.

**Table 5:** Average cost of tests and medicines per episode (in US$)

	National programs		Sub–national programs				
	**Malawi (2010)**	**Senegal (2011)**	**Cameroon (2012)**	**DRC (2012)**	**Sierra Leone (2012)**	**South Sudan (2012)**	**Zambia (2011)**
Fever tested for malaria with RDT (RDT and paracetamol)		1.09					0.65
Malaria	0.76	0.56	0.38	0.47	0.62	0.77	0.85
Pneumonia	0.08	0.04	N.A.	0.10	0.18	0.27	0.56
Diarrhea (ORS and zinc)	0.82	0.09	0.59	0.58	0.51	0.56	0.77

DRC – Democratic Republic of the Congo, RDT – rapid diagnostic test, ORS – oral rehydration solution

### Efficiency

An important ratio for measuring the efficiency of iCCM is the average number of services provided per CHW. This is influenced primarily by the availability of the CHW and the demand for services. In terms of availability, most providers are volunteers and have to also perform income–generating activities (eg, farming or animal husbandry) and many also provide other voluntary health services. Factors influencing the demand for services include the size of the catchment population, the incidence of the illnesses, the distance a person’s home to the place where the CHW is based, perceptions of quality of care, and the availability of medicines.

The average catchment population of children (aged 2–59 months) per CHW differed considerably, with CHWs in Sierra Leone covering an average of 38 children and CHWs in Malawi covering an average of 454 children (**Table 6**). The hours per week that each CHW reported being available for iCCM services also varied – ranging from 4 in Sierra Leone to 49 in the DRC. We recognize that this is very difficult to estimate in most cases, as CHWs do not wait at home for patients. Even though these figures may be somewhat unreliable, they indicate possible differences that are significant, with an average CHW reportedly being available for 48 hours per week for 109 children in Zambia and 16 hours per week for 454 children in Malawi.

**Table 6:** Community health worker (CHW) coverage and efficiency

	National programs		Sub–national programs				
	**Malawi**	**Senegal**	**Cameroon**	**DRC**	**Sierra Leone**	**South Sudan**	**Zambia**
Average number of children 2–59 month per CHW	454	74	60	171	38	180	109
Available CHW hours per week for iCCM	16	11	19	49	4	10	48
Average Number of iCCM Cases seen per CHW (year)	267	22	38	371	117	171	427
Average Number of iCCM Cases seen per CHW (week)	5.0	0.5	0.7	7.1	2.3	3.3	8.2
% of available iCCM time used	15%	2%	12%	10%	74%	85%	11%
Estimated CHW attrition rate	2%	5%	7%	40%	6%	10%	5%
Length of initial CHW training (days)	NA	NA	5	5	6	7	7
CHW remuneration	Yes	Yes	No	No	No	No	No
User fees charged for iCCM services	No	Yes	No	No	No	No	No

DRC – Democratic Republic of the Congo, iCCM – integrated community case management, NA – not available

The average number of cases seen by a CHW ranged from 0.5 per week in Senegal to 8.2 per week in Zambia. In two of the seven countries less than 1 case was seen per week, on average, which raises concerns about the ability of providers to maintain their skill and highlights the importance of hands–on supervision and refresher trainings. For CHWs to maintain their skills, they should see at least 10 cases per month in total, including 1 or 2 pneumonia cases, and they should have good supportive supervision where skills can be regularly assessed [17].

The estimated time spent providing iCCM services ranged from 2% to 85% of the total time they said they were available for iCCM services. In some cases, this probably reflects a high degree of over–estimation of available time reported by CHWs. Reported levels of attrition ranged from 2% to 10% (the rate of 40% reported in the DRC may not be reliable), as shown in the same table, and the higher rates are a concern since experienced, skilled providers may be lost and the cost of training and equipping replacements can be high.

The CHWs were remunerated in the two national programs but not in the five sub–national programs and it is notable that the attrition levels were lower in the national programs. There does not, however, seem to be a relationship between CHW remuneration and the numbers of iCCM cases seen, and a deeper analysis would be needed to explore this due to the contextual factors.

User fees were charged to patients in one of the national programs (Senegal) and the average numbers of iCCM cases seen were low. Again, a deeper analysis would be needed to try to determine if there was a relationship between user fees and utilization levels.

### Additional studies

Other studies have been conducted which add value to the discussion of CHW costs. In particular, an overview of community health workers by Perry et al [18] which examined different CHW models and accompanying models of effectiveness in achieving improved health for communities. Additional insights into the challenges of scaling up iCCM have been provided by Oliver et al [19]. And a study by Seidenberg et al looks at the impact of iCCM on health–seeking behavior in Zambia, in which one of the findings was that iCCM can reduce workload at primary health centers [20]. Further information is expected when the South–African Medical Research Council publishes the results of a UNICEF–funded study of iCCM program costs in 6 African countries.

Studies have also examined how patient costs, such as transport and user fees, can restrict or delay access to health services and can negatively impact on a poor family’s financial situation, as well as indicating how iCCM can alleviate this economic burden by bringing services closer to the family. A study in Uganda showed, for example, that community treatment of malaria and pneumonia resulted in significant cost–saving for rural, poor communities, who would otherwise lose productive time travelling to health facilities [21]. And a study in Pakistan showed that community based management of severe pneumonia can reduce both provider and patient costs while also improving case seeking and treatment compliance [22].

## DISCUSSION

To have maximum impact on child health and mortality, iCCM services should be available within 24 hours of the onset of illness and from a single provider (“one–stop shopping”). This is especially important if there are co–morbidities. If the case is complex or severe, the CHW should be able to refer the case to the nearest health facility and help arrange transport, if necessary.

It is clear that effective iCCM can reduce morbidity and save lives but for the services to be widely accepted and implemented by governments, they must also be affordable and cost–effective. Based on this analysis, there are two main factors that affect this: utilization of services, and management and supervision costs.

The analysis shows that low utilization of iCCM services contributes significantly to high unit costs per service, as fixed supervision and management costs have to be absorbed by fewer cases. The results indicate that iCCM services may have been under–utilized in several of the programs, with less than 20% of the expected number of episodes of illness seen by CHWs and, in some cases, CHWs seeing less than one case per week. Low utilization can also be an issue in terms of quality of care because a provider should see sufficient cases per month to build and retain the necessary experience and skills.

Utilization depends partly on the number and types of service included in the iCCM package. In Cameroon, for example, where pneumonia was not treated as part of iCCM in the year of the study, overall utilization was low and this contributed to a higher average unit cost per service. On the other hand, including other services, such treatment of red eye in Malawi, increased utilization and contributed to the lower average unit cost per service. The degree to which other services can or should be added is, however, an important topic that is beyond the scope of this paper.

Utilization is affected by several other factors such as the size of the catchment population, incidence of illness, CHW access and availability, perceptions of medicine supply and quality of care and perhaps, in some cases, user fees as well. In some of the programs, utilization was low because the catchment populations were small, for example in Sierra Leone where each CHW only covered an average of 38 children. Also, incidence rates were lower in some program areas, such as in Malawi, with 5.3 expected episodes per child per year, compared with 9.6 in Senegal.

The availability of the CHWs does not appear to have been a reason for low utilization, since less than 20% of the reported available time was used for iCCM in 6 of the 7 programs. However, it appears likely that medicine stock–outs have been a factor since this was reported as a problem in several of the studies. The studies did not seek to determine if user fees had an impact on utilization in Senegal, which was the only program that had them, and the results did not indicate any obvious relationship.

The lack of maturity of programs was a possible factor in the low utilization levels seen in three of the sub–national programs that had been running for three years or less. At the 2014 iCCM Symposium in Accra, Ghana, it was noted that it can take at least 3 years before an iCCM program reaches maturity in terms of utilization of services and it may take at least 12 months of implementation at scale (with greater than 80% of CHWs trained) to have higher utilization [3]. Building confidence in the availability and quality of iCCM services can take time, but it seems that active promotion and behavior change activities, including the close involvement of community leaders, can increase demand faster, as has reportedly been the case in the Zambia program, which achieved quite high utilization in less than three years.

As noted above the other key factor in terms of iCCM program costs is management and supervision. In the five sub–national programs, this was over 70% of the total recurrent costs and even though it was much lower in the Malawi national program it was 79% in Senegal, where the national program was run by an international organization. It is understandable that the costs of setting up and running pilot projects can be relatively high and even more so if they are run by local or international organizations. These costs should become much lower in relative terms if the programs are scaled–up and taken over by the government. Nevertheless all programs should aim to minimize these costs while maintaining good support for the CHWs so that the availability and quality of iCCM is optimal. Costs can be minimized, for example, by integrating supervision (eg, covering all community health services, not just iCCM), by combining supervision with outreach visits where additional curative services are provided by the supervisor during the visit, and by using local peer supervisors to supplement professional supervisors.

Another key program cost relates to replacing CHWs who stop working. The cost of training and equipping new CHWs can be significant and is often not budgeted. Moreover, the loss of experienced, knowledgeable CHWs can affect the performance of the program. Attrition rates were 5% or more in all of the programs where CHWs were not remunerated, compared with 2% in Malawi where they were remunerated.

In terms of the impact of remunerating CHWs, there was not enough information to assess whether the additional costs were outweighed by increased utilization and reduced attrition, but that is a possibility that is worth exploring in other studies.

The additional costs of iCCM may be offset to some degree by savings elsewhere in the health system. As mentioned previously, there is some evidence that iCCM can reduce workload at primary health centers, and cost savings should also be achieved by treating cases before they become severe. In addition, there is evidence that iCCM results in savings to families with sick children. Unfortunately, it was not possible to investigate these possibilities in the costing studies.

Finally, it is important to note that iCCM programs were sometimes established as a transition strategy to save lives because primary care facility services were weak. Where this is the case, it should be accepted that iCCM services will be costly and may be unsustainable until primary health facilities are fully functional, taking into account that they need to provide the supervision and support (eg, supplies of medicines) and serve as reliable referral units for severe cases. In small, hard–to–reach communities, however, iCCM will probably be the most cost–effective way to provide services in the long term, even if they are costly.

### Limitations

There were a number of limitations to the studies that could have affected the results and which necessitate the need for caution in interpreting and comparing them. The most significant overall limitation is that the studies were carried out at different time periods in seven very different countries which were selected purposively for reasons other than cross–country comparisons. Other limitations include the following. Some of the sub–national programs only started in 2010, and the use of data from 2011 and 2012 to measure costs and efficiency may be premature as it can sometimes take 3 years before programs reach maturity in terms of utilization of services. The samples of facilities and communities were relatively small and were limited in terms of remote communities. Recurrent costs may be underestimated because of lack of complete information on services provided, such as follow–up visits, numbers of referrals, and treatment of fever which is not diagnosed as malaria or pneumonia. Costs do not include the removal of bottlenecks and other health system strengthening activities or economic costs, such as the value of a voluntary CHW’s time, family out–of–pocket costs or income losses due to treatment seeking. Finally, the measurement of costs and efficiency depend significantly on CHWs’ estimates of time available and time needed for services, and some inaccuracy in these estimates is likely.

### Recommendations

While there are a growing number of studies on iCCM costs, additional analyses are needed to assess the cost–effectiveness of iCCM. Such analyses are important in making a stronger case that iCCM is a worthwhile investment, while simultaneously helping to determine the most affordable ways to provide quality services. There is a need to look at the role of iCCM within the primary health care system, not as an alternative to facility–based or other community services, but as an effective way of providing treatment for key childhood illnesses in hard–to–reach communities. It is important to take into account patient financial and economic costs as well as service provision costs, and to include factors such as timeliness, quality and appropriateness of treatment. There is also a need to look at the costs of removing bottlenecks, including the costs of improving medicine supply and demand generation, as well as the impact of CHW remuneration and the impact of charging patients for services. Supervision and management can be costly element of iCCM, and the cost–effectiveness of strategies to minimize these costs should be explored. Analysis of financing and financial sustainability is also needed, including the use of medicine sales to patients as a way of financing supplies. Finally, system improvements are generally needed to ensure the availability of routine iCCM and CHW service data, which is necessary for in–depth analysis and performance monitoring.

## CONCLUSIONS

The results of this analysis show that in order to be cost–effective and affordable, iCCM programs must be well–utilized and management and supervision must be organized in a way that minimizes cost while ensuring quality of care. This requires the removal of bottlenecks, such as medicine stock–outs, and of any barriers to access. It also requires activities that encourage the utilization of iCCM services such as the promotion of those services and the involvement of community leaders. To minimize the costs of iCCM management and supervision it is important they are an integral part of the routine systems under which, for example, a supervision visit to a community covers multiple health services, not just iCCM.

In some cases, however, it must be accepted that iCCM will not be low–cost even if the CHWs are volunteers. For example, a sub–national iCCM program that is established by an NGO to save lives because facility–based services are weak is likely to be relatively costly until the health system is strengthened. And in the case of small, remote villages, while iCCM is likely to be the most cost–effective way to provide services, it may never be low–cost because of the supervision and supply challenges.
